# Perceptions of brazilian nursing faculty members regarding literacy of human rights related to health in nursing undergraduate programs

**DOI:** 10.1186/s12914-019-0213-7

**Published:** 2019-08-28

**Authors:** Carla Aparecida Arena Ventura, Isabel Amelia Costa Mendes, Simone de Godoy, Laís Fumincelli, Mirella Castellano Souza, Valtuir Duarte Souza Junior

**Affiliations:** 1 University of São Paulo at Ribeirão Preto College of Nursing, WHO Collaborating Centre for Nursing Research Development, Av. Bandeirantes, 3900 Campus Universitário - Bairro Monte Alegre, Ribeirão Preto, SP CEP: 14040-902 Brazil; 2 Federal University of São Carlos (UFSCar), Ribeirão Preto, Sao Paulo Brazil

**Keywords:** Human rights, Human development, Right to health, Global health, Nursing

## Abstract

**Background:**

In the context of global health, the work of nurses is of key importance, given their role as diplomats in global health and as fundamental forces in the construction of global partnerships. This study seeks to identify the understanding and perceptions of Brazilian nursing faculty members regarding literacy of human rights related to health in nursing undergraduate programs.

**Methods:**

Methodological, quantitative and cross-sectional study carried out with nursing faculty members from 20 Brazilian higher education institutions. For the data collection, the Brazilian version of the Basic Core Competencies in Global Health questionnaire was used, available on the website Survey Monkey. In this article, the answers related to the domain “Health as a human right and development resource” were assessed. Descriptive statistics were applied, as well as Cronbach’s alpha coefficient.

**Results:**

In total, 222 questionnaires were completed. As for the domain “Health as a human right and development resource”, Cronbach’s alpha coefficient corresponded to 0.839 for the three domain items. Most of the participants fully agreed on the relevance of the contents related to the theme for nurses’ education.

**Conclusions:**

It is essential that nurses have contact with human rights international instruments that influence implementation of health and health research policies, though this content’s treatment is still incipient in Brazilian nursing programs.

## Background

Globalization based on market rationality, with all the consequences deriving from social injustices and inequalities, leads us to seek other perspectives to analyze human desires and needs [1, 2]. Law, legal thinking and practice committed to human rights drive political, ethical and social agendas that supports the construction of a more comprehensive and inclusive rationality. The idea of the universality of human rights can represent a framework that allows everyone to create conditions for particular conceptions of dignity to become possible [2]. From this perspective, it is important to emphasize that, while human rights derive from social practices, their articulation may also establish new practices. Therefore, the challenge is to seek various forms of guaranteeing human rights, and, among them the right to health, which is the human right focused in this study [3].

There are approximately 20.7 million nurses and midwives globally, constituting the largest group of health care providers [4] in the world. In this context, global nurse citizens must “… think deeply and critically about what is equitable and just, and what will minimize harm to our planet” [5]. Thus, it is important to leverage nurses understanding on global health and human rights related to health issues, educating them to effectively act with healthcare users to locally consolidate the right to health.

The concept of global nursing encompasses the social determinants of health linked to health care for individuals and communities, as well as research, education, leadership, advocacy and policy initiatives [6]. In the light of this concept, it is understood and expected that professionals prepared to act as global nurses have the necessary knowledge and demonstrate respect for human rights and dignity, cultural diversity and ethical attitudes when partnering with other professionals to the good of communities [7, 8].

In 1948, the Universal Declaration of Human Rights, in Article 25, affirmed that “everyone has the right to a standard of living adequate for the health and well-being of himself and of his family, including food, clothing, housing and medical care and necessary social services … “[9], corroborating the existence of the right to health among these other social rights.

In the preamble to the World Health Organization (WHO) Constitution, the Member States declared that, in accordance with the UN Charter, the following principles are basic to the happiness, harmonious relations and security of all peoples: a) Health is a state of complete physical, mental and social well-being and not merely the absence of disease or infirmity; b) The enjoyment of the highest attainable standard of health is one of the fundamental rights of every human being without distinction regarding race, religion, political belief, economic or social condition; c) The health of all peoples is fundamental to the attainment of peace and security and is dependent upon the fullest co-operation of individuals and States; d) The achievement of any State in the promotion and protection of health is of value to all; e) Unequal development in different countries in the promotion of health and control of disease, especially communicable diseases, is a common danger; f) Healthy development of children is of basic importance; the ability to live harmoniously in a changing total environment is essential to such development; g) The extension to all peoples of the benefits of medical, psychological and related knowledge is essential to the fullest attainment of health; h) Informed opinion and active co-operation on the part of the public are of the utmost importance in the improvement of the health of people; i) Governments have a responsibility for the health of their peoples, which can be fulfilled only by the provision of adequate health and social measures [10].

In this scenario, it is stated that the WHO, by associating the concept of health with social and psychological well-being, expresses the idea of the human being in relation to his or her environment. Thus, the right to health not only encompasses the right to healthcare services, but also to an entitlement to services, goods and conditions that are conducive to the realization of the highest attainable standard of physical and mental health [10].

This right was subsequently included in the 1966 UN Covenant on Economic, Social and Cultural Rights (ICESCR) [11], as well as in several other international instruments, such as the Convention on the Elimination of all forms of discrimination against Women (1979), the African Charter on Human and People’s Rights (1981), the Additional Protocol to the American Convention on Human Rights in the area of Economic, Social and Cultural Rights (1988), the Convention on the Rights of the Child (1989), the International Labor Organization (ILO) Convention n. One hundred and sixty-nine concerning Indigenous and Tribal Peoples in Independent Countries (1989), the International Convention on the Protection of the Rights of All Migrant Workers and Members of their Families (1990), the Convention against Torture and other Cruel, Inhuman or Degrading Treatment or Punishment (2002), and the Convention of the Rights of Persons with Disabilities (2006).

In 2001, the UN Committee on Economic, Social and Cultural Rights adopted General Recommendation No. 14, which gives an authoritative interpretation of the right to the highest attainable standard of physical and mental health in Article 12 ICESCR [12]. General Recommendation 14 provides a broad normative interpretation of the right to health, bringing an examination of its scope and meaning. Thus, the right to health is closely related to and dependent upon the realization of other human rights, such as contained in the International Bill of Rights, including the rights to food, housing, work, education, human dignity, life, non-discrimination, equality, privacy, access to information, the prohibition against torture, and the freedoms of association, assembly and movement. These and other rights and freedoms address integral components of the right to health [12].

Social rights, such as the right to health, involve a political aspect related to the functions and duties of the State and are considered rights that can be achieved progressively, as they depend directly on public investment [12–14]. The growing health inequities observed within and among countries demonstrate how distant we are from the true consolidation of these rights in people’s lives [15].

In this context, political, economic, social and cultural processes determine and condition different diseases, increasing the vulnerability of some social groups and enabling the emergence and reemergence of illnesses. These processes increase the interdependence among the different international actors, as health issues literally “cross” borders, becoming global issues.

One of the positive points resulting from globalization has been the expanded awareness of the importance of the impact of this phenomenon on global health and on the range of this concept for college students and professors from health faculties. Particularly in Nursing, it is important to emphasize the position that nurses act as diplomats in global health and as fundamental forces for the construction of global partnerships [16].

Brazilian nursing faculties establish their curriculum frameworks according to the curricular guidelines for undergraduate nursing programs. In this sense, the aim of this study is to identify the perceptions of Brazilian nursing faculty members regarding literacy on human rights related to health in nursing undergraduate programs.

## Method

Quantitative and cross-sectional study involving nursing faculty members from Brazilian Higher Education Institutions (HEIs). Ethical precepts were followed, as approved by the Research Ethics Committee at the University of São Paulo at Ribeirão Preto College of Nursing (CEP / EERP-USP Opinion 1135/2010).

The HEIs were randomly selected from the database of the Secretariat of the Global Network of WHO Collaborating Centres for the Development of Nursing and Midwifery, located at the World Health Organization Collaborating Centre for the Development of Nursing Research, at the University of São Paulo at Ribeirão Preto College of Nursing. Of the 20 HEIs from each region of Brazil, 10 public and 10 private institutions were obtained. In this way, the teachers of the selected institutions were invited, by e-mail, to participate in the research. From these HEIs, participants were invited and characterized according to the inclusion criteria, such as, having a degree in nursing, academic background and activities, a role in Nursing Education, years and experience as Nursing faculty members at undergraduate programs. In addition, the authors identified the types of nursing courses offered at the school of nursing where the participants worked.

For the data collection, the Brazilian version of the Basic Care Competencies in Global Health questionnaire [17] was made available on the Survey Monkey website. This questionnaire consists of 30 competencies in six domains. In this study, the responses related to the domain “Health as a human right and a development resource” were evaluated. The domain refers to the nursing students’ understanding of human rights related to health, which affect the health of individuals and populations, contributing to the effectiveness of advocacy for the health of patients and communities.

Brazilian nursing faculties establish their curriculum frameworks according to the curricular guidelines for undergraduate nursing programs. Furthermore, the questions of this study which were asked to nursing faculty members are based on their perspectives regarding literacy on human rights related to health in nursing undergraduate programs. In other words, participants were asked if they agreed or not that nursing students should be able to: demonstrate basic understanding of the relationship between health and human rights; demonstrate familiarity with the organizations and agreements on human rights related to health care and health research; describe the role of the World Health Organization (WHO) in the articulation between health and human rights, the Universal Declaration of Human Rights, the International Ethical Guidelines for Biomedical Research involving Human Subjects (2002) and the Declaration of Helsinki (2008).

The findings were extracted from the Global Health research database [17]. In order to do so, participants were asked to rate the extent of their agreement as to whether each item was appropriate to nursing students’ training, from 1 “totally disagree” to 4 “totally agree”, but also to indicate if the particular competency was part of the institution’s current curriculum.

For statistical descriptive analysis of the data, the SPSS was used and the frequencies and percentages of each of the domain items were computed, as well as the Cronbach’s alpha coefficient.

## Results

In total, 222 questionnaires were completed. The participating teachers had a mean age of 47.8 years. Of these, 57 (25.7%) had worked in the area less than 10 years, 133 (51.0%) between 10 and 29 years and 32 (14.4%) between 30 and 53 years. Regarding teacher education, 169 (10.8%) held a doctoral degree, 61 (27.4%) a Master’s degree, 17 (7.6%) a *Latu* Sensu post-graduation degree, and 24 (10.8%) a Bachelor’s degree.

In relation to the domain “Health as a human right and development resource”, Cronbach’s alpha coefficient corresponded to 0.839 for the three domain items. The items, their answers and the question whether they are covered in the current curriculum are described in Table 1.

**Table 1:** Description of participants’ answers to the items in the domain Health as a Human Right and Development Resources, which are covered in the current curriculum

Items in the domain health as a human right and development resource		
	n	%
a. Demonstrate basic understanding of the relationship between health and human rights		
I totally disagree	34	15.3
I disagree	1	0.4
I agree	39	17.5
I totally agree	148	66.4
Covered in the current curriculum	131	58.7
b. Demonstrate familiarity with the organizations and agreements on human rights related to health care and health research		
I totally disagree	34	15.3
I disagree	8	3.6
I agree	73	32.7
I totally agree	107	48.0
Covered in the current curriculum	76	34.1
c. Describe the role of the World Health Organization (WHO) in the articulation between health and human rights, the Universal Declaration of Human Rights, the International Ethical Guidelines for Biomedical Research involving Human Subjects (2002) and the Declaration of Helsinki (2008)		
I totally disagree	34	15.3
I disagree	4	1.8
I agree	51	22.9
I totally agree	133	59.6
Covered in the current curriculum	105	47.1

## Discussion

In general, responses to all three questions are “agree” or “totally agree” in over 80% of the respondent’s surveys. However, the percentage who responded “totally agree” declines, indicating a change in the overall support of respondents regarding the items. Among them, the item “Demonstrate basic understanding of the relationship between health and human rights” had the higher percentage of “totally agreement” (66.4%).

Therefore, among the participants, 187 (83.9%) agreed or totally agreed on the importance of the basic understanding of the relationship between health and human rights in the training of nurses in regard to global health. In Brazil, the concept of health as a fundamental human right dates to the 1988 Constitution and was the result of a period of struggles and social movements that culminated in the 8th National Health Conference in 1986. Formerly a right restricted to workers, beginning in1988, health became the right of everyone and the duty of the State [18–21].

The right to health in Brazil can therefore be considered as the result of political struggle, with the active participation of health workers, especially nursing professionals. This history of social mobilization seems to stand out in the minds of professionals, who have demonstrated this awareness by confirming the importance of a basic understanding of the relationship between health and human rights [2, 22].

Health as a social right is thus a structural change the consolidation of which began with the implementation of the Unified Health System (SUS) in 1990. Forms of social and political materialization of health as a true right of citizenship are still necessary, though [23, 24]. In this context of difficulties for the materialization of health as an effective right of citizens in Brazil, 107 (58.7%) research participants confirmed that this content is covered in Brazilian nursing courses. Thus, most of the participants believe that the understanding of health as a human right is fundamental and report that this content, of a political rather than technical nature, is included into their nursing courses.

Regarding the next item of this dimension, 180 (80.7%) participants mentioned agreeing or totally agreeing that nursing students should develop skills to demonstrate familiarity with organizations and agreements dealing with human rights related to health care and research.

In this view, the international movement for the protection of human rights took form at the end of World War II, with the creation of the United Nations (UN) in 1945. The UN Charter contained the relevance of nations seeking to develop cooperative relations and guarantee the human rights of their citizens. It was within the framework of the UN General Assembly that, in 1948, the Universal Declaration of Human Rights was adopted, after which different instruments were developed and approved for the protection of universal human rights, protecting general human rights or specific population groups considered to be more vulnerable, such as children, women and disabled people [9]. Knowing the mission and role of the UN, as well as other international organizations within the so-called UN System (including the World Health Organization (WHO), World Trade Organization (WTO) and International Labor Organization (ILO) permits understanding economic, political and social challenges countries have faced in their international relations, with direct repercussions for their national and local relations.

In this scenario, it is important to be familiar with the insertion of health in the movement to celebrate the Millennium Development Goals (MDGs) implemented from 2000 to 2015 and later in the Sustainable Development Goals (SDGs) in force from 2015 to 2030 [25]. This content is usually addressed within the area called Diplomacy in Global Health [2, 13].

Still in the global health scenario, it is indispensable that nurses understand the complex network established among international guidelines from international organizations, countries and their national health policies, incorporated into their daily work routines [18]. It is also important for nurses to understand the movements of power and cooperation that are intertwined in international relations and historically define the international agenda and national health agendas [4, 13]. Thus, it is relevant for nurses to understand the differences in the implementation of human rights, with focus on the right to health, through health policies and health care in different countries. This knowledge can and should support nurses’ accomplishments of culturally conscious actions in diverse contexts [3, 17].

Records of abuses and violations of the rights of individuals and populations, affecting their health and well-being, are well-known, which is motivation for health professionals to take action to prevent these violations, as well as to assume the responsibility of participating and collaborating actively in the formulation of policies and practices that promote those rights [26]. It is imperative that nurses, as a major professional class in health systems around the world, adopt human rights standards, which is why they need preparation in their school education. Therefore, the institutions that maintain undergraduate nursing courses should be aware of this requirement.

The concept of human rights in patient care is also worth mentioning, as an innovative approach to promote the discourse on human rights and individual and community human rights law in patient care. This approach proposes the abolition of the consumerist view restricted to the delivery of health care. As a preferable alternative, it proposes an approach of health promotion and protection, seeking systematic treatment by health care providers at local, regional and global levels. This applied approach can serve as an instrument to influence policy, protecting the rights of the most vulnerable [27].

Nurses should also have an understanding of historical abuses in conducting research involving human subjects and International Declarations that seek to establish guidelines for the protection of persons participating in scientific research, so that they attach due importance to the protection of these persons in future research they come to coordinate [24, 28]. Moreover, despite considering the content relevant, only 76 (34.1%) participants confirmed its insertion in the curricula of the nursing programs of which they are part.

The last dimension is related to the description of the World Health Organization’s (WHO) role in the articulation between health and human rights as a competency of nurses in global health. [12, 23, 28]. Among the participants, 184 (82.5%) agreed or totally agreed as to their importance, although only 105 (47.1%) affirmed that this content is included in their nursing programs.

As stated earlier, the insertion of historical aspects of the creation of the UN and the WHO (as the technical organization in the field of health in the world) in the content of nursing courses would allow nurses to have a more critical and comprehensive view of national health policies [29]. In this sense, the WHO plays a crucial role in shaping international health law, acting in a decentralized manner and addressing the different characteristics of national health systems [4]. Among the policies established by the WHO since the 1980s, the policy Health for All in the year 2000 stands out and currently the policy focuses on universal health coverage [30, 31].

In general, the study adds support for the idea that nursing faculty recognize the importance of linking health and human rights, supplementing other research results which also valued and emphasized this linkage [3, 32].

## Conclusion

For effective implementation of the right to health, it is of crucial importance that an understanding of this norm be treated as a fundamental global health competency for nursing. Thus, in addition to the work of international organizations, it is fundamental that nurses have contact with international instruments that, even without enforcement characteristics, influence the development and implementation of health policies and health research. In contrast, this sort of content still seems to be treated in a rather incipient way in Brazilian nursing programs.

This study’s focus is grounded in the opinions of faculty members about global health contents related to the human right to health in the nursing curricula. Future studies could focus on undergraduate students in order to have a more complete picture of undergraduate teaching in the area of global health and human rights related to health literacy. In sum, this study is limited to the identification of the support by nursing faculty regarding the inclusion of this content in the nursing undergraduate programs.

## Data Availability

The datasets generated and analyzed during the current study are not publicly available. We are not able to share the data from our participants, because these information and permission were not included in the Informed Consent for this study.

## Abbreviations

HEIs: Brazilian Higher Education Institutions
ICESCR: International Covenant on Economic, Social and Cultural Rights
ILO: International Labor Organization
MDGs: Millennium Development Goals
SDGs: Sustainable Development Goals
SUS: Unified Health System
UN: United Nations
WHO: World Health Organization
WTO: World Trade Organization
